# Heat Shock Protein 60 in Hepatocellular Carcinoma: Insights and Perspectives

**DOI:** 10.3389/fmolb.2020.00060

**Published:** 2020-04-15

**Authors:** Abdullah Hoter, Sandra Rizk, Hassan Y. Naim

**Affiliations:** ^1^Department of Physiological Chemistry, University of Veterinary Medicine Hannover, Hanover, Germany; ^2^Department of Biochemistry and Chemistry of Nutrition, Faculty of Veterinary Medicine, Cairo University, Giza, Egypt; ^3^Department of Natural Sciences, Lebanese American University, Byblos, Lebanon

**Keywords:** heat shock proteins, hepatocellular carcinoma, cancer therapy, chaperones, therapeutic resistance, chaperonin

## Abstract

Heat shock protein 60 (HSP60) is a mitochondrial chaperone that is implicated in physiological and pathological processes. For instance, it contributes to protein folding and stability, translocation of mitochondrial proteins, and apoptosis. Variations in the expression levels of HSP60 have been correlated to various diseases and cancers, including hepatocellular carcinoma (HCC). Unlike other HSPs which clearly increase in some cancers, data about HSP60 levels in HCC are controversial and difficult to interpret. In the current review, we summarize and simplify the current knowledge about the role of HSP60 in HCC. In addition, we highlight the possibility of its targeting, using chemical compounds and/or genetic tools for treatment of HCC.

## Introduction

**FIGURE 1:**
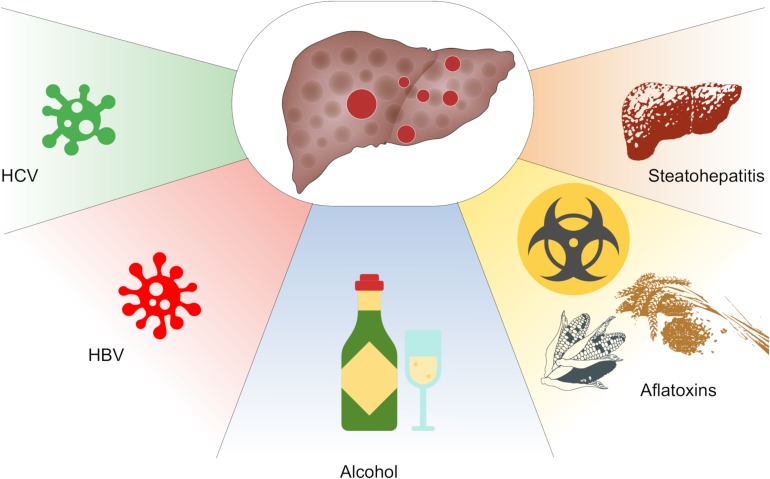
Key factors contributing to hepatocellular carcinoma (HCC). These include chronic infections with infections with hepatitis B (HBV) or hepatitis C (HCV) virus, alcohol abuse, consumption of aflatoxins and non-alcoholic steatohepatitis (NASH).

Hepatocellular carcinoma (HCC) is a common cancer of the liver with worldwide incidence and high mortalities. According to 2018 statistics, HCC is estimated to be the sixth frequently diagnosed cancer and the fourth main cause of death globally (Bray et al., 2018). Notably, the morbidity and mortality rates in men are two–threefold greater than in women. Moreover, HCC represents the most common cancer in 13 geographically diverse nations including countries from Africa like Egypt, Gambia, and Guinea as well as countries from Eastern and South-Eastern Asia such as Mongolia, Cambodia, and Vietnam. Some reports highlight the spread of the disease among developed countries despite the advances in vaccine development (Stasi et al., 2016; Ghouri et al., 2017). Several risk factors lead to HCC development and contribute to its complex pathogenesis such as chronic viral infections including hepatitis B virus (HBV) and hepatitis C virus (HCV), alcoholic cirrhosis, non-alcoholic steatohepatitis (NASH), exposure to chemical carcinogens, and ingestion of aflatoxin-contaminated foods (Fujiwara et al., 2018) (Figure 1).

Heat shock proteins (HSPs) are a set of evolutionarily conserved molecules which exist in almost all living organisms (Lindquist and Craig, 1988; Jäättelä, 1999; Sørensen et al., 2003; Chen et al., 2018). Generally, these numerous proteins are classified into families and subfamilies of distinct molecular masses that range from about 10 to110 kDa. The families of HSPs are grouped into large HSPs such as HSP110 and glucose-regulated protein 170 (GRP170), small HSPs (sHSPs) besides HSP40 (DNAJ), chaperonin, or heat shock protein 60 (HSP60), HSP70, and HSP90 (Kampinga et al., 2009; Chatterjee and Burns, 2017). Members of HSPs are commonly known to function as molecular chaperones assisting in the folding of newly synthesized as well as misfolded proteins. In addition, they are involved in the elimination of terminally misfolded proteins through their targeting to cellular proteolytic machineries (Hartl et al., 2011; Hipp et al., 2019).

HSP60 (also called Cpn60) is a ubiquitous chaperone present in all mammalian cells and tissues including liver. This HSP performs many physiological functions that are not restricted to its cellular canonical location in the mitochondria (Gupta et al., 2008; Merendino et al., 2010). It supports mitochondrial protein folding and assists in proteolytic degradation of denatured or aberrantly folded proteins in an ATP-dependent fashion (Azem et al., 1995; Henderson et al., 2013). HSP plays numerous physiological roles but can also be pathogenic in various conditions, including cancer and neurodegenerative diseases (Cappello et al., 2008; Bross et al., 2012; Zhou et al., 2018). Recent reports highlight the role and implication of HSP60 in human cancer development and management whereby its targeting has revealed promising therapeutic outcomes (Pace et al., 2013; Li et al., 2014; Meng et al., 2018).

HSP60 has been studied as biomarker for diagnosis and prognosis assessment and as therapeutic agent in a range of diseases, such as gastric (Li et al., 2014), bronchial (Cappello et al., 2005), colorectal (Hamelin et al., 2011) and ovarian cancer (Guo et al., 2019), as well as glioblastoma (Khalil and James, 2007) and esophageal squamous cell carcinoma (Faried et al., 2004). Nevertheless, the role of HSP60 in HCC remains poorly understood. In the current review, we discuss the oncogenic role of HSP60 and its potential as therapeutic target, focusing on HCC. Moreover, we shed the light on the potential of HSP60 targeting as a therapeutic strategy to combat HCC.

## Structural and Organizational Aspects of Human HSP60

Generally, the term HSP60 refers to the mtHSP60 while the cytosolic homolog is known as TRiC/CCT. There is a notion that mtHSP60 arose from bacterial ancestors that were engulfed by early eukaryotic cells to produce the mitochondrial organelle (Hemmingsen et al., 1988). Mechanistically, HSP60 (HSPD1) forms tetradecamers consisting of two stacked heptameric rings with a central cavity that harbors the client protein. Importantly, HSPE1 (the homolog of bacterial GroES and known as HSP10) forms a single heptameric ring that functions as a cap for the HSPD1 assembly. This structural organization is essential for the ATP-dependent functionality of HSP60 in protein folding (Pace et al., 2013; Nisemblat et al., 2015; Okamoto et al., 2015). On the other hand, the cytosolic TRiC/CCT consists of eight subunits encoded by *TCP1* and *CCT2-8* genes and has its own built-in cap system (Leitner et al., 2012). In human, HSP60 is encoded by a gene located on chromosome 2q33.1 (Hansen et al., 2003). hHSP60 resides mostly in the mitochondrial matrix and the outer mitochondrial membrane with potential localization to other extra-mitochondrial sites (Soltys and Gupta, 1999; Gupta et al., 2008). Despite its constitutive expression under physiological conditions, increased levels of HSP60 can be induced following mitochondrial damage or heat stress.

In this manuscript, we use the word expression and its derivatives to indicate presence or quantitative changes of any protein, e.g., Hsp60, indiscriminately, without considering the cause, namely whether they are due to changes in the levels of expression of the pertinent gene, or to post-transcriptional or post-translational mechanisms, or a combination of them. Like most HSPs, hHSP60 is regulated via heat shock response by binding of the heat shock element (HSE) to the specific region on the DNA (Hansen et al., 2003). It should be noted that increasing reports correlate the variant expression of hHSP60 in different cellular compartments as well as biological fluids, including blood and cerebrospinal fluid, to human pathological conditions (Deocaris et al., 2006). Hence, detection and quantitative determination of HSP60 alterations may provide clues for studying disease mechanisms, prognosis, and treatment progress (Nakamura and Minegishi, 2013).

## The Anti-Apoptotic and Oncogenic Roles of HSP60

An interesting activity of HSP60 in mammalian cells is its contribution to apoptosis regulation. Early studies in the leukemic Jurkat T cell line revealed that HSP60 and its associated chaperone HSP10 form a complex with caspase-3 leading to its maturation. This observation suggested a potential chaperoning activity of HSP60 toward caspase-3 (Samali et al., 1999; Xanthoudakis et al., 1999). In addition, other studies showed that HSP60 was expressed on the surface of murine lymphoma cells (Sapozhnikov et al., 1999). Moreover, HSP60 has been linked to tumor cell apoptosis in a process that involves increased surface expression of HSP60 and subsequent stimulation of anti-tumor immune responses (Feng et al., 2001). On the other hand, increased expression of HSP60 in cardiac myocytes has been found to inhibit apoptosis indicating a significant yet complex role of HSP60 in the apoptotic machinery of tumor cells (Henderson et al., 2013). These findings in tumor and non-tumor cells raised many questions whether HSP60 is an anti- or pro-apoptotic protein (Henderson et al., 2013). Importantly, the previous study that included many *in vitro* apoptotic systems could unravel some mechanistic lines of HSP60 apoptotic action (Chandra et al., 2007). One significant conclusion was that the cytosolic accumulation of HSP60 is a common process during apoptosis regardless of its mitochondrial release and its pro-survival or pro-apoptotic behavior involves differential interactions with caspase-3 (Chandra et al., 2007).

Owing to its anti-apoptotic properties, it is not surprising that HSP60 displays tumorigenic functions. HSP60 supports cancer development via increasing tumor growth, promoting angiogenesis and metastasis, reducing mitochondrial permeability transition, and counteracting apoptosis (Wu et al., 2017). In accordance with these functions, secretion of HSP60 has been described in all investigated tumor cells suggesting a role in tumor growth and dissemination, where the secretion process was independent of cell death (Merendino et al., 2010). Further molecular investigations revealed that pro-carcinogenic effects of HSP60 are due to its ability to enhance cancer cell survival via interacting with and inhibiting the intracellular isoform of clusterin in neuroblastoma cells (Chaiwatanasirikul and Sala, 2011).

**FIGURE 2:**
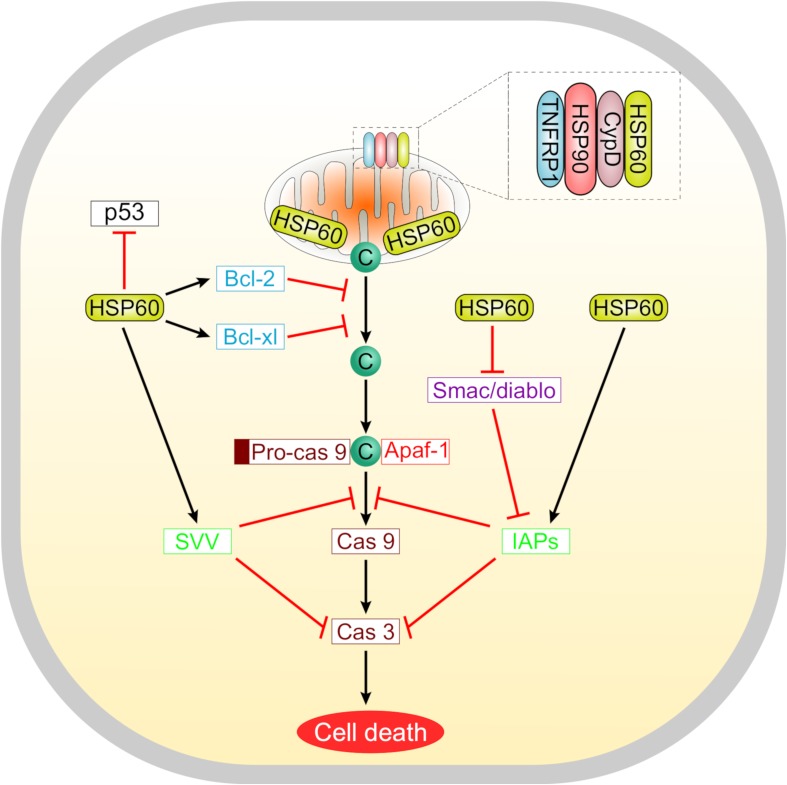
A schematic representation summarizing the roles of HSP60 in regulating tumor cell apoptosis. Suppression of apoptosis by HSP60 is associated with increased levels of anti-apoptotic proteins like Bcl-2, Bcl-xL, and survivin. These molecules counteract the release of cytochrome c from mitochondria. Selectively in tumor cells, HSP60 forms a multichaperone complex with Cyclophilin D (Cyp D) and other chaperones including HSP90 and tumor necrosis factor receptor-associated protein 1 (TNFRP1) to maintain the mitochondrial permeability transition. Oncogenic HSP60 interacts with p53 in tumor cells and suppresses its action. HSP60 also controls the progression of apoptosis via regulating the mitochondrial release of smac/diablo and controlling the function of inhibitor of apoptosis (IAP) family proteins that inhibit caspases.

Suppression of apoptosis by HSP60 is concomitant with overexpression of the anti-apoptotic proteins Bcl-2, Bcl-xL, and survivin, maintenance of the mitochondrial transmembrane potential, and inhibition of caspase 3 activation (Deocaris et al., 2006). Cytosolic HSP60 inhibits the translocation of the pro-apoptotic protein Bax into the mitochondria, hence promoting cell survival (Xanthoudakis et al., 1999; Lianos et al., 2015). Furthermore, the anti-apoptotic actions of HSP60 involve its interaction with several molecules including the mitochondrial HSP70, survivin, and p53. HSP60 is also a potent regulator of the mitochondrial permeability transition which is meditated through a multichaperone complex comprising HSP60, HSP90, and tumor necrosis factor receptor-associated protein-1 (TNFRP1), particularly assembled in tumors but not in normal cells (Ghosh et al., 2010; Rodríguez et al., 2016) (Figure 2). In tumor cells, the anti-apoptotic HSP60 has been found to interact with cyclophilin D in the mitochondrial permeability transition pore where subsequent disruption of this interaction altered the mitochondrial permeability transition, stimulated caspase-dependent apoptosis, and led to suppression of tumor cell growth (Ghosh et al., 2010).

Interestingly, inhibition of HSP60 could enhance the tumor suppressive activity of insulin-like growth factor binding protein 7 (IGFBP7) in colorectal cancer cells (Ruan et al., 2010). In human cancer HeLa cells, cytosolic HSP60 has been shown to support nuclear factor-kB (NF-kB)-dependent survival through binding and regulating the activity of IkB kinase (IKK) (Chun et al., 2010). Moreover, HSP60 knockdown in ovarian cancer cells inhibits tumor progression by breaking mitochondrial proteostasis, and inactivating the mTOR pathway (Guo et al., 2019). With regard to cancer metastasis, HSP60 has been described as a key chaperone that promotes metastatic phenotypes both *in vitro* and *in vivo* (Tsai et al., 2009). This effect is attributed to a significant interaction between HSP60 and β-catenin resulting in overexpression of β-catenin and enhancement of its transcriptional activity (Tsai et al., 2009).

Upregulation of HSP60 has been implicated in cervical cancer progression and prognosis (Hwang et al., 2009). Likewise, expression of HSP60 has been linked to the progression of prostate cancer (Castilla et al., 2010; Hoter et al., 2019). In ovarian cancer, the HSP60 transcript levels were variable in cancerous tissues. However, several reports linked high HSP60 expression to advanced ovarian cancer stages highlighting its prognostic value (Kimura et al., 1993; Hjerpe et al., 2013; Hoter and Naim, 2019). In breast cancer, autoantibodies against HSP60 have been reported to be elevated and their levels were remarkably associated with cancer grade (Hamrita et al., 2008). Sera obtained from patients with colorectal cancer showed increased concentrations of HSP60 that were associated with advanced stages of colorectal cancer (Hamelin et al., 2011). These findings strengthen the notion of utilizing HSP60 as a diagnostic and prognostic marker in several types of cancers.

## HSP60 is a Key Player in Major Etiologies of HCC

In-depth analyses of the etiologies leading to HCC reveal that HSP60 plays a yet unclear role in diverse causalities of HCC development. HSP60 is implicated in the predisposing factors of HCC including HBV, HCV, and alcoholic hepatitis as described in the next section.

## HSP60 in Hepatitis B Virus (HBV) Infection

HBV infection is a major risk factor for the development of HCC worldwide. Chronic infection with HBV predisposes patients with cirrhotic liver or those devoid of hepatic cirrhotic lesions to HCC (Xu et al., 2014). Notably, the central burden of the HCC (85%) is noticed in the HBV endemic regions (Niu and Hann, 2017). Initial studies demonstrated that HSP60 interacts with human HBV polymerase leading to its maturation into its active form (Park and Jung, 2001; Park et al., 2002). Moreover, other studies showed that the transcriptional transactivating protein HBx of HBV form a complex with HSP60 and HSP70 suggesting their contribution to virus-mediated liver oncogenesis (Zhang et al., 2005). In line with previous findings, comparative proteomic analysis of liver HepG2.2.15 cell line revealed that HSP60 forms, together with HSP70 and HSP90, a chaperone complex that is suggested to influence HBV production and secretion (Liu et al., 2009). Furthermore, targeted therapy toward HSP60 using antisense oligonucleotide greatly interferes with HBV replication (Park et al., 2003).

## HSP60 in Hepatitis C Virus (HCV) Infection

Similar to its role in HBV infection, HSP60 has been found to mediate HCV infection in several aspects. HSP60 has been demonstrated to interact with the N-terminal domain of the core structural protein of HCV which is involved in HCV chronic infection-associated liver diseases (Kang et al., 2009). The HSP60–HCV interaction has been found to mediate the production of reactive oxygen species (ROS) and regulate apoptosis. Moreover, high expression of HSP60 in human liver cells expressing the HCV core protein could protect against TNF-α induced cell lethality via reduction of ROS production (Kang et al., 2009).

## HSP60 in Alcoholic Hepatitis

Initial studies showed that HSP60 levels were increased in liver tissues of patients with acute alcoholic liver. Additionally, raised circulating anti-HSP60 IgA levels have been reported. These findings suggest that overall upregulation of HSP60 in tissues and serum might be one of the pathogenic mechanisms leading to the development and progression of liver damage in alcoholic hepatitis (Koskinas et al., 1993). In accordance with these results, other research groups reported a strong association between IgA levels against HSP60 and patients with acute alcoholic hepatitis (Winrow et al., 1994).

## HSP60 in HCC

Early studies showed that the expression of HSP60 was highly increased in chronic active hepatitis with preferential accumulation in areas of inflammatory infiltrates (Lohse et al., 1993). Several proteomic and immunohistochemical analyses revealed upregulation of a multitude of chaperones including HSP60 in HCC (Lim et al., 2002, 2005; Kim et al., 2003). Recent reports showed that both blood and urine mRNA levels of HSP60 were significantly higher in HCC post-HCV patients compared with those having cirrhosis or healthy controls (Abd El-Salam et al., 2017). Moreover, proteomic analysis of HCC tumor associated antigens has identified HSP60 as an important HCC marker. Further serological investigations revealed that HSP60 could be detected not only in sera of HCC patients but also in patients with chronic hepatitis and liver cirrhosis (Looi et al., 2008). With reference to recent meta-analysis performed on patients with digestive cancers, HSP60 has been considered an advanced biomarker for HCC (Chen et al., 2019).

Molecular studies revealed that the redox status of HSP60 is crucial in the hepatocyte growth factor (HGF)-induced ERK phosphorylation and HepG2 cell migration (Lin et al., 2016). Interestingly, there are discrepancies among the recent studies concerning the expression levels and molecular roles of HSP60 in HCC. For instance, one study reported a reduction in HSP60 expression levels in HCC tissues compared to the peritumor tissues (Zhang et al., 2016). Further analyses performed by this group revealed that lowered HSP60 levels were significantly linked to the tumor differentiation grade whereby downregulation of HSP60 was associated with poor prognosis. Additionally, increased HSP60 expression cancer/pericancer (C/P) ratio was associated with enhanced overall survival as confirmed in a cohort study of 107 HCC patients (Zhang et al., 2016). At the molecular level, HSP60 was found to stimulate the differentiation of BEL-7402 and SMMC7721 and inhibit the invasion of BEL-7402 and SK-Hep-1 HCC cell lines. Furthermore, *in vivo* experiments have proven that ectopic expression of HSP60 exerts significant anti-metastatic activity following inoculation into hepatic tissue of nude mice (Zhang et al., 2016). Another biological function reported was that overexpression of HSP60 has been associated with mitochondrial biogenesis as confirmed by its direct correlation to the levels of mitochondrial biogenesis markers cytochrome c oxidase subunit (COX4) and mitochondrial DNA (mtDNA) (Zhang et al., 2016). These findings are in contrast with those obtained by another group as summarized in Table 1 (Huang et al., 2018).

**TABLE 1:** Highlights of the two contradicting studies concerning HSP60 roles in HCC.

HSP60 main function	HSP60 functions as tumor suppressor of HCC^a^	HSP60 supports the development and progression of HCC^b^
HSP60 levels	Both mRNA and protein levels are reduced in tumor tissues of HCC patients compared with peritumor tissues	In liver tissues of HCC patients, high levels of HSP60 were found in 56.6% of specimens compared to 40.0% with reduced HSP60 levels
Clinical correlation	HSP60 expression ratio in cancerous and pericancerous tissues of HCC patients [expressed as cancer/pericancer (C/P) ratio] is associated with overall survival. Low ratio is linked to poor overall survival and vice versa	No significant correlation between the quantitative variations of HSP60 expressed as paired tumor/non-tumor ratio (T/N) ratio and the clinical outcomes Only elevated HSP60 expression levels in non-cancerous tissues were associated with shorter overall survival. Moreover, there exists a positive correlation between HSP60 expression in non- cancerous tissues and macrovascular invasion, high tumor grades, and large tumor size
*In vivo* findings	Ectopic expression of Hsp60 in the left hepatic lobes of nude mice stimulated the differentiation and suppressed both intrahepatic and lung metastasis	Targeting HSP60 in mice xenograft models by jetPEI/HSP60-shRNA revealed significant reduction in both tumor size and weight compared to the control group
Molecular roles of HSP60 in HCC cells	HSP60 induced differentiation	Its role in differentiation was not experimentally investigated
	HSP60 inhibited invasion and metastasis	Its role in metastasis was not experimentally investigated
	HSP60 promotes mitochondrial biogenesis	No significant alterations in mitochondrial mass upon HSP60-silencing in HCC cells
	No effect on proliferation	HSP60 silencing resulted in marked suppression of cell growth and proliferation
	No effect on apoptosis	HSP60 silencing destabilized survivin and promoted cell death
Conclusion	Hsp60 exerts a tumor suppressor function, and might be utilized as a potential therapeutic target in the treatment of HCC	HSP60 can serve as prognostic marker as well as a therapeutic target for HCC

*^*a,b*^Studies performed by Zhang et al. (2016) and Huang et al. (2018), respectively.*

## Targeting HSP60 in HCC

Recent reports highlight the perspectives of targeting HSP60 as a future tool for cancer treatment (Nakamura and Minegishi, 2013; Cappello et al., 2014; Meng et al., 2018). In fact, HSP60 inhibition has been implemented as a therapeutic strategy in various types of cancers including melanoma (Kamm et al., 2019), pancreatic cancer (Zhou et al., 2018), and ovarian cancer (Guo et al., 2019). However, few reports concerned its targeting in HCC. Although one previous study (Zhang et al., 2016), discussed in the previous section, reported contradicting roles for HSP60 in terms of apoptosis and proliferation to what is commonly known about this chaperone in most cancer types, different findings have been reported (Huang et al., 2018) (Table 1). In experiments aiming at elucidating the potential of HSP60 inhibition for HCC treatment, this group has revealed significant enhancement of cell apoptosis, reduction of cell proliferation, and downregulation of survivin upon silencing of HSP60 in two HCC cell lines (Huang et al., 2018). Interestingly, these apoptotic and anti-proliferative effects were minimal or neglected in HSP60-silenced healthy hepatocytes (Huang et al., 2018). In line with these *in vitro* experiments, suppression of HSP60 in hepatoma xenograft mice models using jet polyethylenimine mediated shHSP60 delivery systems (PEI/shHSP60 complexes) resulted in significant reduction of tumor volumes and weights in the shHSP60 group compared to the control group (Huang et al., 2018). On the other hand, the clinical outcomes relative to HSP60 expression were different in the respective investigating groups. While one group showed a correlation between Hsp60 expression and clinico-pathological characteristics of HCC patients, the other group revealed no significant correlation. Their conclusion was attributed to relative high expression of HS60 in both cancerous and corresponding non-cancerous hepatic tissues in HCC patients.

## Inhibitors of HSP60

Compared with other HSPs such as HSP90 and HSP70, few compounds have been known to target HSP60 in the oncology field (Nakamura and Minegishi, 2013). Recent reports have shed the light on the potential HSP60 inhibitors and their usage for cancer therapeutic purposes (Meng et al., 2018). According to their mechanism of action, HSP60 inhibitors have been classified into type I inhibitors and type II inhibitors. Type I inhibitors act by blocking the ATP binding site of HSP60 and subsequently interfering with ATP hydrolysis and folding activities, whereas Type II compounds function through covalent interaction with specific cysteine residues within the HSP60 molecule. Nevertheless, full understanding of the exact mechanisms of HSP60 inhibitors is still lacking (Meng et al., 2018). Table 2 provides an overview about the known HSP60 inhibitors which might be used in future HCC studies or combination therapies for cancer treatment.

**TABLE 2:** Summary of known HSP60 inhibitors^a^.

Class	Inhibitor	Effect	References
	Mizoribine	Binds to the HSP60 ATPase domain and inhibits the chaperoning activity of Hsp60–Hsp10 complex	Itoh et al., 1999; Tanabe et al., 2012
Natural products	Epolactaene	Inhibits the chaperone activity of HSP60 with yet unclear mechanism	Nagumo et al., 2005; Meng et al., 2018
	ETB (tert-butyl ester of epolactaene)	Interacts with Cys442 of HSP60 leading to potential allosteric modulation of the ATP binding pocket	Nagumo et al., 2005
	Myrtucommulone A (MC)	Interacts directly with HSP60 leading to aggregation and misfolding of cancer related proteins	Wiechmann et al., 2017
	Stephacidin B	Performs anticancer activities	Qian-Cutrone et al., 2002; Wulff et al., 2007
	Avrainvillamide	Anticancer activities	Fenical et al., 2000
Synthetic compounds	*O*-carboranylphenoxyacetanilide	Binds to HSP60 and inhibits the hypoxia-inducible factor (HIF) activation	Ban et al., 2010
	Gold (III) porphyrin complexes such as A prototype gold (III) complex [Au(TPP)Cl] (10)	Despite its poorly understood mechanisms, it suppresses HSP60 and performs significant anticancer activities	Lease et al., 2013; Teo et al., 2014; Hu et al., 2016

*^*a*^Nakamura and Minegishi, 2013; Meng et al., 2018.*

## Conclusion and Perspectives

The enigma of HSP60 as a tumor suppressor or oncogenic molecule in HCC still exists. If we consider previous studies on the benefits of HSP60 targeting in variant cancer types such as in melanoma and ovarian cancer, we encourage further investigations aiming at finding ways to treat HCC by using drugs targeted at HSP60, to inhibit it if oncogenic or to potentiate it if anti-tumor. Since many HSP60 inhibitors are currently known and the list is still growing, several *in vitro* and *in vivo* studies are primarily and extensively needed to evaluate these inhibitors and their efficacy in HCC treatment regimens.

## Author Contributions

AH, SR, and HN conceived the review topic. AH wrote the review draft and designed the figures. SR and HN edited and revised the review draft. All authors approved the final version of the review.

## Conflict of Interest

The authors declare that the research was conducted in the absence of any commercial or financial relationships that could be construed as a potential conflict of interest.

## References

[B1] Abd El-Salam F. M. El-Sharqawy E. H. El-feky H. M. Mohammed S. A. Edres A. M. (2017). Heat shock protein 60 and chromatin assembly factor-1 mRNA levels in hepatitis C virus-related hepatocellular carcinoma and clinical significance. *Int. J. Res. Med. Sci.* 5:965 10.18203/2320-6012.ijrms20170644

[B2] Azem A. Diamant S. Kessel M. Weiss C. Goloubinoff P. (1995). The protein-folding activity of chaperonins correlates with the symmetric GroEL14(GroES7)2 heterooligomer. *Proc. Natl. Acad. Sci. U.S.A.* 92 12021–12025. 10.1073/pnas.92.26.12021 8618836 PMC40288

[B3] Ban H. S. Shimizu K. Minegishi H. Nakamura H. (2010). Identification of HSP60 as a primary target of o-carboranylphenoxyacetanilide, an HIF-1alpha inhibitor. *J. Am. Chem. Soc.* 132 11870–11871. 10.1021/ja104739t 20695501

[B4] Bray F. Ferlay J. Soerjomataram I. Siegel R. L. Torre L. A. Jemal A. (2018). Global cancer statistics 2018: GLOBOCAN estimates of incidence and mortality worldwide for 36 cancers in 185 countries. *CA. Cancer J. Clin.* 68 394–424. 10.3322/caac.21492 30207593

[B5] Bross P. Magnoni R. Bie A. S. (2012). Molecular chaperone disorders: defective Hsp60 in neurodegeneration. *Curr. Top. Med. Chem.* 12 2491–2503. 10.2174/1568026611212220005 23339303

[B6] Cappello F. Conway De Macario E. Marasà L. Zummo G. Macario A. J. L. (2008). Hsp60 expression, new locations, functions and perspectives for cancer diagnosis and therapy. *Cancer Biol. Ther.* 7 801–809. 10.4161/cbt.7.6.6281 18497565

[B7] Cappello F. Di Stefano A. D’Anna S. E. Donner C. F. Zummo G. (2005). Immunopositivity of heat shock protein 60 as a biomarker of bronchial carcinogenesis. *Lancet. Oncol.* 6:816 10.1016/S1470-2045(05)70393-70394 16198989

[B8] Cappello F. Marino Gammazza A. Palumbo Piccionello A. Campanella C. Pace A. Conway de Macario E. (2014). Hsp60 chaperonopathies and chaperonotherapy: targets and agents. *Expert Opin. Ther. Targets* 18 185–208. 10.1517/14728222.2014.856417 24286280

[B9] Castilla C. Congregado B. Conde J. M. Medina R. Torrubia F. J. Japón M. A. (2010). Immunohistochemical expression of Hsp60 correlates with tumor progression and hormone resistance in prostate cancer. *Urology* 76 1017.e1–1017.e6. 10.1016/j.urology.2010.05.045 20708221

[B10] Chaiwatanasirikul K.-A. Sala A. (2011). The tumour-suppressive function of CLU is explained by its localisation and interaction with HSP60. *Cell Death Dis.* 2:e219. 10.1038/cddis.2011.99 22012253 PMC3219095

[B11] Chandra D. Choy G. Tang D. G. (2007). Cytosolic accumulation of HSP60 during apoptosis with or without apparent mitochondrial release: evidence that its pro-apoptotic or pro-survival functions involve differential interactions with caspase-3. *J. Biol. Chem.* 282 31289–31301. 10.1074/jbc.M702777200 17823127

[B12] Chatterjee S. Burns T. F. (2017). Targeting heat shock proteins in cancer: a promising therapeutic approach. *Int. J. Mol. Sci.* 18:2017. 10.3390/ijms18091978 28914774 PMC5618627

[B13] Chen B. Feder M. E. Kang L. (2018). Evolution of heat-shock protein expression underlying adaptive responses to environmental stress. *Mol. Ecol.* 27 3040–3054. 10.1111/mec.14769 29920826

[B14] Chen Y. Li X. Shao S. (2019). The clinical value of HSP60 in digestive system cancers: a systematic review and meta-analysis. *Clin. Lab.* 65:523. 10.7754/Clin.Lab.2019.190523 31625354

[B15] Chun J. N. Choi B. Lee K. W. Lee D. J. Kang D. H. Lee J. Y. (2010). Cytosolic Hsp60 is involved in the NF-kappaB-dependent survival of cancer cells via IKK regulation. *PLoS One* 5:e9422. 10.1371/journal.pone.0009422 20351780 PMC2843631

[B16] Deocaris C. C. Kaul S. C. Wadhwa R. (2006). On the brotherhood of the mitochondrial chaperones mortalin and heat shock protein 60. *Cell Stress Chaperon.* 11 116–128. 10.1379/csc-144r.1 16817317 PMC1484513

[B17] Faried A. Sohda M. Nakajima M. Miyazaki T. Kato H. Kuwano H. (2004). Expression of heat-shock protein Hsp60 correlated with the apoptotic index and patient prognosis in human oesophageal squamous cell carcinoma. *Eur. J. Cancer* 40 2804–2811. 10.1016/j.ejca.2004.08.013 15571964

[B18] Feng H. Zeng Y. Whitesell L. Katsanis E. (2001). Stressed apoptotic tumor cells express heat shock proteins and elicit tumor-specific immunity. *Blood* 97 3505–3512. 10.1182/blood.v97.11.3505 11369644

[B19] Fenical W. Jensen P. R. Cheng X. C. (2000). *Avrainvillamide, a Cytotoxic Marine Natural Product, And Derivatives There of US Patent.* Available at: http://patft.uspto.gov/netacgi/nph-Parser?d=PALL&p=1&u=%2Fnetahtml%2FPTO%2Fsrchnum.htm&r=1&f=G&l=50&s1=6066635.PN.&OS=PN/6066635&RS=PN/6066635 (accessed January 20, 2020).

[B20] Fujiwara N. Friedman S. L. Goossens N. Hoshida Y. (2018). Risk factors and prevention of hepatocellular carcinoma in the era of precision medicine. *J. Hepatol.* 68 526–549. 10.1016/j.jhep.2017.09.016 28989095 PMC5818315

[B21] Ghosh J. C. Siegelin M. D. Dohi T. Altieri D. C. (2010). Heat shock protein 60 regulation of the mitochondrial permeability transition pore in tumor cells. *Cancer Res.* 70 8988–8993. 10.1158/0008-5472.CAN-10-2225 20978188 PMC2982903

[B22] Ghouri Y. A. Mian I. Rowe J. H. (2017). Review of hepatocellular carcinoma: epidemiology, etiology, and carcinogenesis. *J. Carcinog.* 16:1. 10.4103/jcar.JCar_9_16 28694740 PMC5490340

[B23] Guo J. Li X. Zhang W. Chen Y. Zhu S. Chen L. (2019). HSP60-regulated mitochondrial proteostasis and protein translation promote tumor growth of ovarian cancer. *Sci. Rep.* 9:12628 10.1038/s41598-019-48992-48997 PMC6718431 31477750

[B24] Gupta R. S. Ramachandra N. B. Bowes T. Singh B. (2008). Unusual cellular disposition of the mitochondrial molecular chaperones Hsp60, Hsp70 and Hsp10. *Novartis Found. Symp.* 291 59–68. 10.1002/9780470754030.ch5 18575266

[B25] Hamelin C. Cornut E. Poirier F. Pons S. Beaulieu C. Charrier J.-P. (2011). Identification and verification of heat shock protein 60 as a potential serum marker for colorectal cancer. *FEBS J.* 278 4845–4859. 10.1111/j.1742-4658.2011.08385.x 21973086 PMC3265716

[B26] Hamrita B. Chahed K. Kabbage M. Guillier C. L. Trimeche M. Chaïeb A. (2008). Identification of tumor antigens that elicit a humoral immune response in breast cancer patients’ sera by serological proteome analysis (SERPA). *Clin. Chim. Acta.* 393 95–102. 10.1016/j.cca.2008.03.017 18424265

[B27] Hansen J. J. Bross P. Westergaard M. Nielsen M. N. Eiberg H. Børglum A. D. (2003). Genomic structure of the human mitochondrial chaperonin genes: HSP60 and HSP10 are localised head to head on chromosome 2 separated by a bidirectional promoter. *Hum. Genet.* 112 71–77. 10.1007/s00439-002-0837-839 12483302

[B28] Hartl F. U. Bracher A. Hayer-Hartl M. (2011). Molecular chaperones in protein folding and proteostasis. *Nature* 475 324–332. 10.1038/nature10317 21776078

[B29] Hemmingsen S. M. Woolford C. van der Vies S. M. Tilly K. Dennis D. T. Georgopoulos C. P. (1988). Homologous plant and bacterial proteins chaperone oligomeric protein assembly. *Nature* 333 330–334. 10.1038/333330a0 2897629

[B30] Henderson B. Fares M. A. Lund P. A. (2013). Chaperonin 60: a paradoxical, evolutionarily conserved protein family with multiple moonlighting functions. *Biol. Rev. Camb. Philos. Soc.* 88 955–987. 10.1111/brv.12037 23551966

[B31] Hipp M. S. Kasturi P. Hartl F. U. (2019). The proteostasis network and its decline in ageing. *Nat. Rev. Mol. Cell Biol.* 20 421–435. 10.1038/s41580-019-0101-y 30733602

[B32] Hjerpe E. Egyhazi S. Carlson J. Stolt M. F. Schedvins K. Johansson H. (2013). HSP60 predicts survival in advanced serous ovarian cancer. *Int. J. Gynecol. Cancer* 23 448–455. 10.1097/IGC.0b013e318284308b 23429486

[B33] Hoter A. Naim H. Y. (2019). Heat shock proteins and ovarian cancer: important roles and therapeutic opportunities. *Cancers* 11:1389. 10.3390/cancers11091389 31540420 PMC6769485

[B34] Hoter A. Rizk S. Naim H. Y. (2019). The multiple roles and therapeutic potential of molecular chaperones in prostate cancer. *Cancers* 11:1194. 10.3390/cancers11081194 31426412 PMC6721600

[B35] Hu D. Liu Y. Lai Y.-T. Tong K.-C. Fung Y.-M. Lok C.-N. (2016). Anticancer gold(III) porphyrins target mitochondrial chaperone Hsp60. *Angew. Chem. Int. Ed. Engl.* 55 1387–1391. 10.1002/anie.201509612 26663758

[B36] Huang Y.-H. Lin K.-H. Yu J.-S. Wu T.-J. Lee W.-C. Chao C. C. K. (2018). Targeting HSP60 by subcutaneous injections of jetPEI/HSP60-shRNA destabilizes *Cytoplasmic survivin* and inhibits hepatocellular carcinoma growth. *Mol. Carcinog.* 57 1087–1101. 10.1002/mc.22827 29672920

[B37] Hwang Y. J. Lee S. P. Kim S. Y. Choi Y. H. Kim M. J. Lee C. H. (2009). Expression of heat shock protein 60 kDa is upregulated in cervical cancer. *Yonsei Med. J.* 50 399–406. 10.3349/ymj.2009.50.3.399 19568603 PMC2703764

[B38] Itoh H. Komatsuda A. Wakui H. Miura A. B. Tashima Y. (1999). Mammalian HSP60 is a major target for an immunosuppressant mizoribine. *J. Biol. Chem.* 274 35147–35151. 10.1074/jbc.274.49.35147 10574997

[B39] Jäättelä M. (1999). Heat shock proteins as cellular lifeguards. *Ann. Med.* 31 261–271. 10.3109/07853899908995889 10480757

[B40] Kamm A. Przychodzeń P. Kuban–Jankowska A. Marino Gammazza A. Cappello F. Daca A. (2019). 2-Methoxyestradiol and its combination with a natural compound, ferulic acid, induces melanoma cell death via downregulation of Hsp60 and Hsp90. *J. Oncol.* 2019 1–12. 10.1155/2019/9293416 32082378 PMC7012217

[B41] Kampinga H. H. Hageman J. Vos M. J. Kubota H. Tanguay R. M. Bruford E. (2009). Guidelines for the nomenclature of the human heat shock proteins. *Cell Stress Chaperon.* 14 105–111. 10.1007/s12192-008-0068-67 PMC2673902 18663603

[B42] Kang S.-M. Kim S.-J. Kim J.-H. Lee W. Kim G.-W. Lee K.-H. (2009). Interaction of hepatitis C virus core protein with Hsp60 triggers the production of reactive oxygen species and enhances TNF-alpha-mediated apoptosis. *Cancer Lett.* 279 230–237. 10.1016/j.canlet.2009.02.003 19264393

[B43] Khalil A. A. James P. (2007). Biomarker discovery: a proteomic approach for brain cancer profiling. *Cancer Sci.* 98 201–213. 10.1111/j.1349-7006.2007.00374.x 17233837 PMC11158801

[B44] Kim W. Oe Lim S. Kim J.-S. Ryu Y. H. Byeon J.-Y. Kim H.-J. (2003). Comparison of proteome between hepatitis B virus- and hepatitis C virus-associated hepatocellular carcinoma. *Clin. Cancer Res.* 9 5493–5500. 14654528

[B45] Kimura E. Enns R. E. Alcaraz J. E. Arboleda J. Slamon D. J. Howell S. B. (1993). Correlation of the survival of ovarian cancer patients with mRNA expression of the 60-kD heat-shock protein HSP-60. *J. Clin. Oncol.* 11 891–898. 10.1200/JCO.1993.11.5.891 8098058

[B46] Koskinas J. Winrow V. R. Bird G. L. A. Lau J. Y. N. Portmann B. C. Blake D. R. (1993). Hepatic 60-kD heat-shock protein responses in alcoholic hepatitis. *Hepatology* 17 1047–1051. 10.1002/hep.1840170617 8514253

[B47] Lease N. Vasilevski V. Carreira M. de Almeida A. Sanaú M. Hirva P. (2013). Potential anticancer heterometallic Fe-Au and Fe-Pd agents: initial mechanistic insights. *J. Med. Chem.* 56 5806–5818. 10.1021/jm4007615 23786413 PMC3880617

[B48] Leitner A. Joachimiak L. A. Bracher A. Mönkemeyer L. Walzthoeni T. Chen B. (2012). The molecular architecture of the eukaryotic chaperonin TRiC/CCT. *Structure* 20 814–825. 10.1016/j.str.2012.03.007 22503819 PMC3350567

[B49] Li X. Xu Q. Fu X. Luo W. (2014). Heat shock protein 60 overexpression is associated with the progression and prognosis in gastric cancer. *PLoS One* 9:e107507. 10.1371/journal.pone.0107507 25207654 PMC4160299

[B50] Lianos G. D. Alexiou G. A. Mangano A. Mangano A. Rausei S. Boni L. (2015). The role of heat shock proteins in cancer. *Cancer Lett.* 360 114–118. 10.1016/j.canlet.2015.02.026 25721081

[B51] Lim S.-O. Park S.-G. Yoo J.-H. Park Y.-M. Kim H. Jang K. (2005). Expression of heat shock proteins (HSP27, HSP60, HSP70, HSP90, GRP78, GRP94) in hepatitis B virus-related hepatocellular carcinomas and dysplastic nodules. *World J. Gastroenterol.* 11 2072–2079. 10.3748/wjg.v11.i14.2072 15810071 PMC4305774

[B52] Lim S. O. Park S.-J. Kim W. Park S. G. Kim H.-J. Kim Y.-I. (2002). Proteome analysis of hepatocellular carcinoma. *Biochem. Biophys. Res. Commun.* 291 1031–1037. 10.1006/bbrc.2002.6547 11866469

[B53] Lin C.-Y. Hu C.-T. Cheng C.-C. Lee M.-C. Pan S.-M. Lin T.-Y. (2016). Oxidation of heat shock protein 60 and protein disulfide isomerase activates ERK and migration of human hepatocellular carcinoma HepG2. *Oncotarget* 7 11067–11082. 10.18632/oncotarget.7093 26840563 PMC4905458

[B54] Lindquist S. Craig E. A. (1988). The heat-shock proteins. *Annu. Rev. Genet.* 22 631–677. 10.1146/annurev.ge.22.120188.003215 2853609

[B55] Liu K. Qian L. Wang J. Li W. Deng X. Chen X. (2009). Two-dimensional blue native/SDS-PAGE analysis reveals heat shock protein chaperone machinery involved in hepatitis B virus production in HepG2.2.15 cells. *Mol. Cell. Proteom.* 8 495–505. 10.1074/mcp.M800250-MCP200 18984579 PMC2649812

[B56] Lohse A. W. Dienes H. P. Herkel J. Hermann E. van Eden W. Meyer zum Büschenfelde K. (1993). Expression of the 60 kDa heat shock protein in normal and inflamed liver. *J. Hepatol.* 19 159–166. 10.1016/s0168-8278(05)80189-80188 7905491

[B57] Looi K. S. Nakayasu E. S. Diaz R. A. de Tan E. M. Almeida I. C. Zhang J.-Y. (2008). Using proteomic approach to identify tumor-associated antigens as markers in hepatocellular carcinoma. *J. Proteome Res.* 7 4004–4012. 10.1021/pr800273h 18672925 PMC2680441

[B58] Meng Q. Li B. X. Xiao X. (2018). Toward developing chemical modulators of hsp60 as potential therapeutics. *Front. Mol. Biosci.* 5:35 10.3389/fmolb.2018.00035 PMC5920047 29732373

[B59] Merendino A. M. Bucchieri F. Campanella C. Marcianò V. Ribbene A. David S. (2010). Hsp60 is actively secreted by human tumor cells. *PLoS One* 5:e9247. 10.1371/journal.pone.0009247 20169074 PMC2821922

[B60] Nagumo Y. Kakeya H. Shoji M. Hayashi Y. Dohmae N. Osada H. (2005). Epolactaene binds human Hsp60 Cys442 resulting in the inhibition of chaperone activity. *Biochem. J.* 387 835–840. 10.1042/BJ20041355 15603555 PMC1135015

[B61] Nakamura H. Minegishi H. (2013). HSP60 as a drug target. *Curr. Pharm. Des.* 19 441–451. 10.2174/13816128130310 22920899

[B62] Nisemblat S. Yaniv O. Parnas A. Frolow F. Azem A. (2015). Crystal structure of the human mitochondrial chaperonin symmetrical football complex. *Proc. Natl. Acad. Sci. U.S.A.* 112 6044–6049. 10.1073/pnas.1411718112 25918392 PMC4434751

[B63] Niu B. Hann H.-W. (2017). “Hepatitis B virus–related hepatocellular carcinoma: carcinogenesis, prevention, and treatment,” in *Updates in Liver Cancer*, Vol. 13 ed. Abdeldayem H. (London: IntechOpen), 10.5772/65424, Available online at: https://www.intechopen.com/books/updates-in-liver-cancer

[B64] Okamoto T. Ishida R. Yamamoto H. Tanabe-Ishida M. Haga A. Takahashi H. (2015). Functional structure and physiological functions of mammalian wild-type HSP60. *Arch. Biochem. Biophys.* 586 10–19. 10.1016/j.abb.2015.09.022 26427351

[B65] Pace A. Barone G. Lauria A. Martorana A. Piccionello A. P. Pierro P. (2013). Hsp60, a novel target for antitumor therapy: structure-function features and prospective drugs design. *Curr. Pharm. Des.* 19 2757–2764. 10.2174/1381612811319150011 23092316

[B66] Park S. G. Jung G. (2001). Human hepatitis B virus polymerase interacts with the molecular chaperonin Hsp60. *J. Virol.* 75 6962–6968. 10.1128/JVI.75.15.6962-6968.2001 11435576 PMC114424

[B67] Park S. G. Lee S. M. Jung G. (2003). Antisense oligodeoxynucleotides targeted against molecular chaperonin Hsp60 block human hepatitis B virus replication. *J. Biol. Chem.* 278 39851–39857. 10.1074/jbc.M301618200 12869561

[B68] Park S. G. Lim S. O. Jung G. (2002). Binding site analysis of human HBV pol for molecular chaperonin, hsp60. *Virology* 298 116–123. 10.1006/viro.2002.1496 12093179

[B69] Qian-Cutrone J. Huang S. Shu Y.-Z. Vyas D. Fairchild C. Menendez A. (2002). Stephacidin A and B: two structurally novel, selective inhibitors of the testosterone-dependent prostate LNCaP cells. *J. Am. Chem. Soc.* 124 14556–14557. 10.1021/ja028538n 12465964

[B70] Rodríguez M. E. Cogno I. S. Milla Sanabria L. S. Morán Y. S. Rivarola V. A. (2016). Heat shock proteins in the context of photodynamic therapy: autophagy, apoptosis and immunogenic cell death. *Photochem. Photobiol. Sci.* 15 1090–1102. 10.1039/c6pp00097e 27471925

[B71] Ruan W. Wang Y. Ma Y. Xing X. Lin J. Cui J. (2010). HSP60, a protein downregulated by IGFBP7 in colorectal carcinoma. *J. Exp. Clin. Cancer Res.* 29 41. 10.1186/1756-9966-29-41 20433702 PMC2873425

[B72] Samali A. Cai J. Zhivotovsky B. Jones D. P. Orrenius S. (1999). Presence of a pre-apoptotic complex of pro-caspase-3, Hsp60 and Hsp10 in the mitochondrial fraction of jurkat cells. *EMBO J.* 18 2040–2048. 10.1093/emboj/18.8.2040 10205158 PMC1171288

[B73] Sapozhnikov A. M. Ponomarev E. D. Tarasenko T. N. Telford W. G. (1999). Spontaneous apoptosis and expression of cell surface heat-shock proteins in cultured EL-4 lymphoma cells. *Cell Prolif.* 32 363–378. 10.1111/j.1365-2184.1999.tb01354.x 10646688 PMC6495567

[B74] Soltys B. J. Gupta R. S. (1999). Mitochondrial-matrix proteins at unexpected locations: are they exported? *Trends Biochem. Sci.* 24 174–177. 10.1016/s0968-0004(99)01390-1390 10322429

[B75] Sørensen J. G. Kristensen T. N. Loeschcke V. (2003). The evolutionary and ecological role of heat shock proteins. *Ecol. Lett.* 6 1025–1037. 10.1046/j.1461-0248.2003.00528.x

[B76] Stasi C. Silvestri C. Voller F. Cipriani F. (2016). The epidemiological changes of HCV and HBV infections in the era of new antiviral therapies and the anti-HBV vaccine. *J. Infect. Public Health* 9 389–395. 10.1016/j.jiph.2015.05.004 26148849

[B77] Tanabe M. Ishida R. Izuhara F. Komatsuda A. Wakui H. Sawada K. (2012). The ATPase activity of molecular chaperone HSP60 is inhibited by immunosuppressant mizoribine. *Am. J. Mol. Biol.* 2 93–102. 10.4236/ajmb.2012.22010

[B78] Teo R. D. Gray H. B. Lim P. Termini J. Domeshek E. Gross Z. (2014). A cytotoxic and cytostatic gold(III) corrole. *Chem. Commun.* 50 13789–13792. 10.1039/c4cc06577h 25252099

[B79] Tsai Y.-P. Yang M.-H. Huang C.-H. Chang S.-Y. Chen P.-M. Liu C.-J. (2009). Interaction between HSP60 and beta-catenin promotes metastasis. *Carcinogenesis* 30 1049–1057. 10.1093/carcin/bgp087 19369584

[B80] Wiechmann K. Müller H. König S. Wielsch N. Svatoš A. Jauch J. (2017). Mitochondrial chaperonin HSP60 is the apoptosis-related target for myrtucommulone. *Cell Chem. Biol.* 24 614.e–623.e. 10.1016/j.chembiol.2017.04.008 28457707

[B81] Winrow V. R. Bird G. L. Koskinas J. Blake D. R. Williams R. Alexander G. J. M. (1994). Circulating IgA antibody against a 65 kDa heat shock protein in acute alcoholic hepatitis. *J. Hepatol.* 20 359–363. 10.1016/s0168-8278(94)80008-80001 8014447

[B82] Wu J. Liu T. Rios Z. Mei Q. Lin X. Cao S. (2017). Heat Shock Proteins and Cancer. *Trends Pharmacol. Sci.* 38 226–256. 10.1016/j.tips.2016.11.009 28012700

[B83] Wulff J. E. Herzon S. B. Siegrist R. Myers A. G. (2007). Evidence for the rapid conversion of stephacidin B into the electrophilic monomer avrainvillamide in cell culture. *J. Am. Chem. Soc.* 129 4898–4899. 10.1021/ja0690971 17397160 PMC3175819

[B84] Xanthoudakis S. Roy S. Rasper D. Hennessey T. Aubin Y. Cassady R. (1999). Hsp60 accelerates the maturation of pro-caspase-3 by upstream activator proteases during apoptosis. *EMBO J.* 18 2049–2056. 10.1093/emboj/18.8.2049 10205159 PMC1171289

[B85] Xu H.-Z. Liu Y.-P. Guleng B. Ren J.-L. (2014). Hepatitis B virus-related hepatocellular carcinoma: pathogenic mechanisms and novel therapeutic interventions. *Gastrointest. Tumor.* 1 135–145. 10.1159/000365307 26676160 PMC4645579

[B86] Zhang J. Zhou X. Chang H. Huang X. Guo X. Du X. (2016). Hsp60 exerts a tumor suppressor function by inducing cell differentiation and inhibiting invasion in hepatocellular carcinoma. *Oncotarget* 7 68976–68989. 10.18632/oncotarget.12185 27677587 PMC5356605

[B87] Zhang S. M. Sun D. C. Lou S. Bo X. C. Lu Z. Qian X. H. (2005). HBx protein of hepatitis B virus (HBV) can form complex with mitochondrial HSP60 and HSP70. *Arch. Virol.* 150 1579–1590. 10.1007/s00705-005-0521-521 15789261

[B88] Zhou C. Sun H. Zheng C. Gao J. Fu Q. Hu N. (2018). Oncogenic HSP60 regulates mitochondrial oxidative phosphorylation to support Erk1/2 activation during pancreatic cancer cell growth. *Cell Death Dis.* 9:161. 10.1038/s41419-017-0196-z 29415987 PMC5833694

